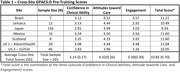# Clinician Attitudes and Confidence on the Detection and Management of Cognitive Impairment: Results from the Davos Alzheimer’s Collaborative Early Detection Program

**DOI:** 10.1002/alz.087959

**Published:** 2025-01-09

**Authors:** Phyllis Barkman Ferrell, James F. Murray, Daniel E Ball, Otelo Corrêa dos Santos Filho, Marcilea Dias de Sá Paiva Lima, Ishtar Govia, Janelle Robinson, Hisatomo Kowa, Mariana López‐Ortega, Alison McKean, Wendy Chambers, Magda R. Baksh, Valeria Baldivieso, Deanna R Willis, Nicole R Fowler, Katherine J. Selzler

**Affiliations:** ^1^ Davos Alzheimer’s Collaborative, Wayne, PA USA; ^2^ Human Aging Center, State University of Rio de Janeiro, Rio de Janeiro, Rio de Janeiro Brazil; ^3^ Municipal Health Department of Volta Redonda, Rio de Janeiro Brazil; ^4^ Jamaica Mental Health Advocacy Network, Kingston Jamaica; ^5^ CUNY Graduate Center, New York, NY USA; ^6^ Kobe University, Kobe Japan; ^7^ Instituto Nacional de Geriatría (National Institute of Geriatric), Mexico City, EM Mexico; ^8^ Brain Health Scotland, Edinburgh United Kingdom; ^9^ NHS Dumfries and Galloway, Dumfries United Kingdom; ^10^ AdventHealth, Orlando, FL USA; ^11^ Indiana University School of Medicine, Indianapolis, IN USA

## Abstract

**Background:**

Early symptoms of cognitive impairment are frequently undetected. The Davos Alzheimer’s Collaborative System Preparedness (DAC‐SP) Early Detection program implemented a digital cognitive assessment (DCA) in primary care and other non‐specialty settings to increase the rate of detection of cognitive impairment.

**Methods:**

The DAC‐SP Early Detection program was initiated in 2021 in seven healthcare systems across six countries. Clinicians were trained on a DCA, including positive tests for cognitive impairment and diagnostic assessment. Prior to training or naïve to implementing a DCA in clinical practice, clinicians’ attitudes and confidence in diagnosis and managing dementia were assessed using the validated General Practitioners Attitude and Confidence Scale for Dementia (GPACS‐D), which is comprised of 15 items in three subscales: Confidence in Clinical Abilities, Attitude towards Care, and Engagement. Each item is measured on a 5‐point Likert scale (1 = strongly disagree; 5 = strongly agree), and subscale scores are standardized. A total of 265 pre‐training surveys were completed across the 7 sites. The GPACS‐D results were calculated by averaging individual physician results per the validated scoring algorithm. The cross‐site results were calculated by taking an equally weighted average across the seven sites.

**Results:**

The GPACS‐D results across the seven sites are presented in Table 1. Across all sites, baseline attitude towards care was the highest of the three subscales. For most sites, confidence in clinical abilities received the lowest scores, with engagement scores only modestly higher. The total and subscale scores were consistent across sites, supported by the relatively low standard deviation.

**Conclusion:**

The findings from the GPACS‐D total and subscale scores suggest that prior to receiving training on using a digital cognitive assessment for their patient populations, clinicians’ attitudes towards the diagnosis and management of cognitive impairment were similar across the seven sites, and independent of the country in which they practice. Despite positive attitudes toward care, the results suggest that education or training focused more on engagement and confidence may improve early detection and care of patients with cognitive impairment, particularly as new diagnostic and therapeutic options emerge.